# 5G Standalone and 4G Multi-Carrier Network-in-a-Box Using a Software Defined Radio Framework †

**DOI:** 10.3390/s21165653

**Published:** 2021-08-22

**Authors:** Karolis Kiela, Marijan Jurgo, Vytautas Macaitis, Romualdas Navickas

**Affiliations:** 1 Lime Microsystems, Surrey Tech Centre, Occam Road, The Surrey Research Park, Guildford GU2 7YG, Surrey, UK; k.kiela@limemicro.com (K.K.); m.jurgo@limemicro.com (M.J.); v.macaitis@limemicro.com (V.M.); 2 Micro and Nanoelectronics Systems Design and Research Laboratory, Vilnius Gediminas Technical University, 10257 Vilnius, Lithuania

**Keywords:** RAN, RRH, SDR, NIB, 5G, 4G, framework, standalone, multicarrier, aggregation

## Abstract

In this work, an open Radio Access Network (RAN), compatible, scalable and highly flexible Software Defined Radio (SDR)-based Remote Radio Head (RRH) framework is proposed and designed. Such framework can be used to implement flexible wideband radio solutions, which can be deployed in any region, have common radio management features, and support various channel bandwidths. Moreover, it enables easier access for researchers to nonsimulated cellular networks, reduce system development time, provide test and measurement capabilities, and support existing and emerging wireless communication technologies. The performance of the proposed SDR framework is validated by creating a Network-in-a-Box (NIB) that can operate in multiband multicarrier 4G or 5G standalone (SA) configurations, with an output power of up to 33 dBm. Measurement results show, that the 4G and 5G NIB can achieve, respectively, up to 883 Mbps and 765 Mbps downlink data transfer speeds for a 100 MHz aggregated bandwidth. However, if six carriers are used in the 4G NIB, 1062 Mbps downlink data transfer speed can be achieved. When single user equipment (UE) is used, maximum uplink data transfer speed is 65.8 Mbps and 92.6 Mbps in case of 4G and 5G, respectively. The average packet latency in case of 5G is up to 45.1% lower than 4G. CPU load by the eNodeB and gNodeB is proportional to occupied bandwidth, but under the same aggregated DL bandwidth conditions, gNodeB load on the CPU is lower. Moreover, if only 1 UE is active, under same aggregated bandwidth conditions, the EPC CPU load is up to four times lower than the 5GC.

## 1. Introduction

### 1.1. Background

The mobile communication industry has seen a dramatic growth in the last two decades since various social communication and entertainment services shifted to mobile technology-oriented user equipment (UE). While earlier generations of cellular technology (such as 4G) focused on connectivity, 5G expands on this by delivering connected experiences from the cloud to customers [1]. Because 4G is well in the commercial deployment stage, 5G and the future mobile technologies (such as 6G) have become a global research and development topic [2], where low power consumption, massive equipment connectivity, ultra-low latency, security, services, deployment and management cost are key challenges [1,2].

Compared to 4G technology, 5G networks are more software-driven and can utilise new networking principles such as Software-Defined Networking (SDN) architectures, virtualisation, Multi-access Edge Computing (MEC) to achieve dynamic network management and enhanced Mobile Broadband (eMBB) [1,2,3]. MEC enables SDN and virtualisation technology to flexibly and quickly manage resources, which can be brought from the remote cloud to the wireless edge in the proximity of the UE [4]. MEC in 5G moves the data plane from the cloud as close to the UE as possible, and, as a result, latency, data security, reliability, and stability are improved, and control level of provided services becomes higher [4,5]. 

As opposed to mobile broadband, current low power, low bandwidth scenarios, namely Internet of Things (IoT), usually use 4G technology-based LTE-M and NB-IoT Low Power Wide Area Network (LPWAN) radio standards. Other common wireless standards used in IoT technologies are Zigbee, LoRaWAN, IEEE 802.11ah (HaLow) and 802.11af (White-Fi) [6]. On the other hand, 5G will be at the core of the emerging IoT revolution, with support for Ultra-Reliable Low Latency Communications (URLLC), massive Machine-Type Communications (mMTC), Device-to-Device (D2D), Machine-to-Machine (M2M) communication [1,2,7]. 5G is also expected to play a major role in Intelligent Transport Systems (ITS), smart city applications, smart industrial software, smart homes, and implementation of many high-end, mission-critical IoT initiatives [1].

Another advantage of 5G is the utilisation of network slicing and open Radio Access Network (RAN) technologies, which can reduce deployment costs of new generation cellular technology due to reuse of the infrastructure, what in turn can also open up new market opportunities [3,8,9]. Open RAN allows to decouple the radio hardware from the radio functionality. The Baseband Unit (BBU) can run in the cloud (cloud RAN or C-RAN) or in a commercial off-the-shelf (COTS) local server (virtual RAN or vRAN), while having a stable, high-speed connection with the Remote Radio Head (RRH). By shifting BBU to the cloud or COTS server, C-RAN and vRAN allow to split the functions of a Radio Access Technology (RAT) between dedicated hardware and software instances [10]. If implementation level of the software communication stack is highest—that is, physical layer 1 (L1) is implemented in BBU—it is possible to have direct access to samples from RRH, which now acts as the radio front-end only. While implementing L1 related functions in RRH could reduce transmission bandwidth between RRH and BBU, this would decrease flexibility in network upgrades and would be less convenient for multicell collaborative signal processing [11].

### 1.2. Motivation

To achieve the desired flexibility, scalability, and resource reusability inherent in the open RAN technology, RRH hardware must also meet the same criteria. One of the widely developed and promising technologies is a Software Defined Radio (SDR). SDR-based transceivers can be used to implement a flexible wideband radio solutions, which can be deployed in any region, have common radio management features, support various channel bandwidths and can be used as O-RAN compliant white-box hardware [12]. O-RAN, not to be confused with open RAN, refers to the O-RAN Alliance, which is a name for a specification group defining next generation RAN infrastructures. SDR-based RRH usually utilises SDR Radio Frequency (RF) transceivers to transmit and receive data in a wide frequency range, usually implemented as a single integrated circuit (IC) [13]. In addition to the RF transceiver, SDR-based RRHs usually use Field-Programmable Gate Array (FPGA) ICs to implement functionalities of the physical layer using digital signal processing algorithms implemented in an embedded system with the aid of a specific software [14,15]. 

Just a few years ago, the majority of researchers had no access to actual cellular networks, and even when they did, it was limited to individual network components or functionalities [3]. Now, an ever-increasing number of frameworks and projects of the RAN-oriented software stack are becoming available. On the other hand, availability of the SDR-based RRH frameworks is still limited. In survey [3], the reviewed RAN-oriented software stack to SDR RRH hardware provider ratio was 3.25:1. In another survey [16], the available software versus hardware framework ratio was 2.75:1. 

To meet open RAN goals of reducing vendor dependency for RAN hardware, it is necessary not only to develop open RAN and 5G Core (5GC) software, but also to develop SDR-based frameworks, and, on their basis, complete Network-in-a-box (NIB) solutions that reduce system development time, provide test and measurement capabilities, and support existing and emerging wireless communication technologies [6,17].

### 1.3. Related Work

Related work to our research can be broken into three categories:

1. RAN and NIB related surveys.
2. RAN simulation-based performance evaluation.
3. Performance evaluations of networks using NIB testbeds.

In this subsection, an overview of most prominent works from each category will be presented.

An extensive survey on RAN architectures for 5G mobile networks regarding energy consumption, operations expenditure, resource allocation, spectrum efficiency, system architecture, and network performance is presented in [11]. The paper also investigates key technologies of the 5G systems, such as MEC, SDN, and network slicing; and major 5G RATs such as millimetre wave (mmWave), massive multiple-input and multiple-output (MIMO), D2D, mMTC; and it provides insight into some major research challenges in these fields. 

In [3], a survey on open source 5G RAN and 5GC is presented, in which RAN and core network software, virtualisation and management frameworks are analysed. In addition, this survey analyses SDR support for open-source radio units and 5G testbed that can be used to instantiate software-based 5G networks. 

Software and hardware tools used in NIB solutions are surveyed in [16]. The paper analyses NIB related works regarding to radio access and backhaul technology, use cases, ease of deployment, edge services, network self-organising features, capacity, and Quality of Service (QoS), hardware implementation approach.

5G RAN performance evaluation can be done in two ways: by using simulators [18,19], or by using testbeds containing all the required hardware and software. In [9], 5G deployment scenarios for Standalone (SA) and Nonstandalone (NSA) are evaluated through coverage, power consumption and handover simulation results. The evaluation is done in the 900–3500 MHz RF frequency range, based on a UE using 100 MHz bandwidth. Based on simulation and analysis results, it is summarised that SA outperforms NSA in terms of UE power consumption, network deployment complexity, and cost. 

5G network MEC service simulation for downlink (DL) resource-block occupancy, frame latency at different frame sizes and UE count is presented in [20]. 4G, 5G NSA and 5G SA scenarios are analysed working at 2000 MHz RF frequency range, with a varying number of UEs (up to 40) using 20 MHz bandwidth. The results show, that deploying 5G NSA and especially 5G SA should remove the 4G bottleneck from Quality of Experience (QoE) perspective and add considerable user capacity.

In [21], simulation of vehicle platooning using Cellular Vehicle-to-Everything (C-V2X) and D2D communication is analysed. The results show that D2D allows one to save 73% of DL frequency resources, which results in less energy consumed by the gNodeBs (gNBs).

While 5G performance evaluation using a simulation-based approach is a convenient way to evaluate performance of different services or communications, simulation based results should always be properly validated to ensure that the results obtained with it are credible and match real world deployment scenarios [21]. This requirement cannot always be met, and in certain scenarios it is even not viable. Simulations also do not necessarily consider open RAN solution based on dynamic behaviour of a general-purpose processor (GPP), for example Central Processing Unit’s (CPU) resource allocation. Hence, whenever possible, RAN performance evaluation using NIB-based testbeds should be the preferred choice if this option is available. 

A GPP-based software defined 4G NIB testbed is presented in [22]. Here, the authors compare different full 4G stack solutions, including OpenAirInterface (OAI), Amarisoft and srsLTE. The testbed uses a 6 core, 3.2 GHz processor to implement a 20 MHz bandwidth, frequency division duplex (FDD), single-input and single-output (SISO) transmission mode (TM). Performance evaluation was done with a DL carrier frequency of 2.660 GHz (Band 7) and achieved a maximum data rate of 70, 70, 30 Mbps in DL and 28, 45, 28 Mbps in uplink (UL) when using respectively OAI, Amarisoft and srsLTE RAN and core software. Ettus B210 SDR and Amarisoft proprietary PCIe SDR was used for eNodeB (eNB) radio front-end. In the presented testbed, Amarisoft software performed best in respect to CPU utilisation, maximum radio link throughput and stability over time, and delay. It should be noted that both RAN and core software used separate GPPs.

A 5G NSA and 4G NIB solution using an open-source software stack based on OpenAirInterface (OAI) is presented in [8]. eNB/gNB and Evolved Packet Core (EPC) software is executed on two separate GPPs, while eNB and gNB front ends are based on Ettus B210 (SDR). 5G NSA implementation uses 40 MHz bandwidth and achieves a maximum DL data rate of 30.7 Mbps when operating at Band 78. In 4G mode operating at Band 3 and Band 40 with a bandwidth of 5 and 10 MHz, data speeds of 5.31 and 8.73 Mbps are achieved respectively in DL.

Another 5G NSA and 4G testbed is reported in [23]. It uses Option 3X architecture in a COTS infrastructure with 64 CPUs and 128 GigaByte (GB) Random Access Memory (RAM), where 16 CPUs are actively used for the core network. 5G NSA implementation uses 100 MHz bandwidth and achieves a maximum DL and UL data rate of 885 and 92 Mbps, respectively, when operating at Band 78. In 4G mode operating at Band 3 with a bandwidth of 80 MHz, data speeds of 420 Mbps and 87 Mbps are achieved in DL and UL, respectively. 

It should be noted that none of the reviewed NIBs use high-power radio Front End Modules (FEM), so they cannot be used in larger scale testbed evaluation scenarios, for example, network rollouts. Moreover, the reviewed 5G NSA NIB solutions do not specify 5G radio link time division duplex (TDD) and TM configurations, which hinders performance comparison. 

Due to limited flexibility of available SDR-based RRH frameworks, available NIB solutions are limited to single band operation in both 4G and 5G. Hence, solutions for expansion of the existing system capacity in existing infrastructure, for example usage of higher-level Carrier aggregation (CA), are not sufficiently explored and compared with 5G technology.

In our previous work [6], we have presented structure of V2X–IoT framework for ITS applications. However, it was mostly theoretical work based on findings from our previous reports. In this work, our previously proposed structure is adapted and an open RAN compatible, scalable and highly flexible SDR-based RRH framework is proposed and designed. The performance of the proposed SDR framework is validated by creating an NIB that can operate in multiband multicarrier 4G or 5G SA configurations. To the best of the authors’ knowledge, none of the previous NIB related works provide a bandwidth-to-bandwidth 4G to 5G SA performance evaluation. This article consists of four chapters. In the second chapter, structure of SDR framework is presented. In the third chapter, structure of NIB is presented. In the fourth chapter, NIB performance evaluation results are reported. The final chapter concludes the presentation.

## 2. Structure of Software Defined Radio Framework

### 2.1. Structure of the Software Defined Radio Framework Hardware

**Figure 1:**
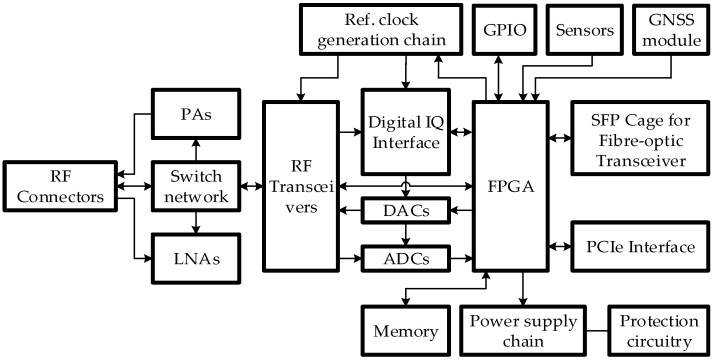
A structure of the proposed SDR framework hardware [6].

As was shown in our previous works [6,17], frameworks and/or development kits that are currently available on the market are most suited for applications with a small selection of pre-defined standards. Such frameworks are not flexible enough in situations where there is a need to implement a solution which is based on a new or emerging application/standard. There is a need for a sufficiently flexible framework that can be used to evaluate current and to develop new communication networks or their architectures and concurrently verify them at hardware and software level. Therefore, SDR-based framework hardware structure is proposed whose simplified block diagram is shown in Figure 1.

This structure is described in detail in [6]. The main feature of such framework is the capability to reconfigure software and hardware, what enables the framework to be used in many deployment and verification scenarios. The software flexibility is provided by the FPGA, which can be configured in such a way to accelerate solution verification; i.e., it has reconfigurable hardware solutions for specific functions, such as image processing or filtering.

The most important function of the FPGA is the transmission and pre-processing of the digital signal to and from the RF transceivers. Analog-to-digital converter (ADC) and the digital-to-analog converter (DAC) are used to convert analog signals to digital and vice-versa. Clock signal generator is used to generate clock frequency for the ADC and DAC. This generator uses a reference clock frequency generation module with a temperature stabilised, voltage-controlled quartz crystal oscillator, all of which are also controlled by the FPGA. The quartz oscillator can be tuned by varying its control voltage. This allows implementing frequency synchronization by using an external or Global Navigation Satellite System (GNSS) module reference signal.

The FPGA is connected using several input/output (I/O) interfaces that are used for firmware programming, control and sending/receiving data: general-purpose I/O (GPIO); SFP cage for fibre-optic transceiver, PCIe interface. Moreover, memory modules, various sensors (temperature, acceleration, humidity, etc.) are connected to the FPGA, which can be employed if SDR framework is used not only in mobile communication equipment, but also for other purposes, such as application in intelligent transport systems (ITS).

GNSS module is used for positioning and tracking. Moreover, it is used for time or frequency synchronisation.

FPGA is also used to manage power supply, which is needed to implement flexible extension of the framework by connecting additional modules with different power supply requirements.

The flexibility of the radio components is implemented through a set of SDR RF transceivers. These transceivers should be broadband and support at least two wireless standards operating in frequency-division duplex simultaneously. The RF transceivers are connected to the reference clock signal generation module, which provides the reference signals needed for the internal high frequency synthesisers. The RF transceivers are coupled with an external band filter module, which allows implementation of specific, or stringent, filtering requirements. High frequency input/output ports of the RF transceivers can be connected to additional transmitter power amplifier (PA) and/or receiver low noise amplifier (LNA). The switching of the high frequency I/O is accomplished by utilising RF switches.

### 2.2. Structure of the Software Defined Radio Based Framework Software

**Figure 2:**
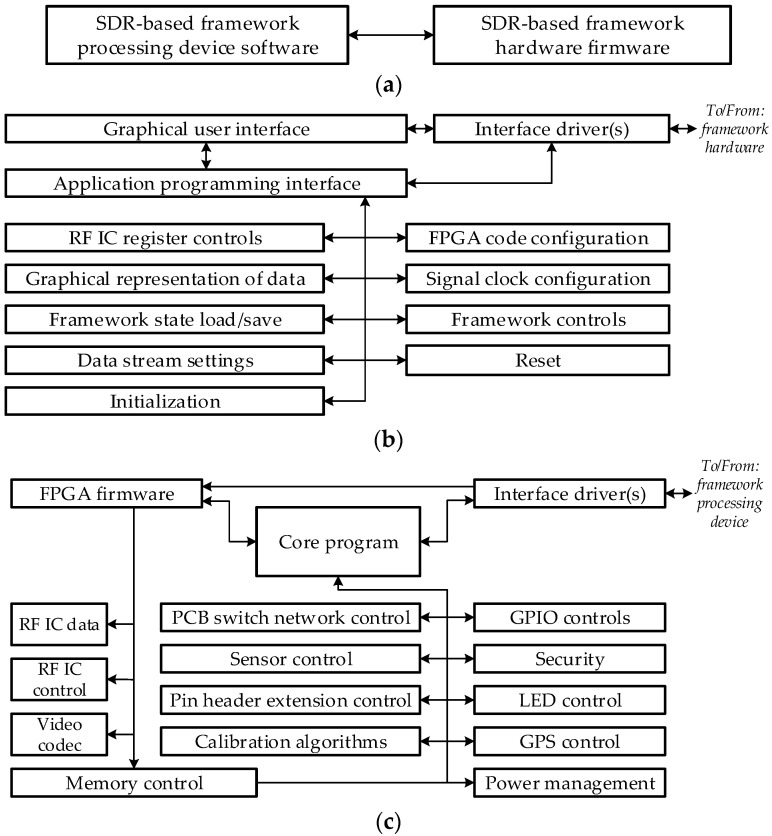
Structure of the software for the software defined radio-based framework [6]: (**a**) highest level of abstraction; (**b**) structure of the processing device software; (**c**) structure of the firmware.

Software of our proposed framework is also described in [6]. At the highest level of abstraction, a software of the framework is divided into two interconnected blocks as shown in Figure 2a. Processing device software is designed to simplify the management of the framework and can be used to process, visualise (render), send and receive data from the hardware. This part of the program has a graphical user interface, is used to load or update firmware and may be integrated with other software.

Hardware firmware implements low level of abstraction functions and functions such as digital filtering, interpolation and decimation, signal framing, timestamping, creating, and managing communication settings between the processing device and hardware, and other functions.

Because the hardware of the proposed framework is very versatile, software also has to be platform-independent for simplified integration with other existing systems or systems which are in development, including mobile devices. Structure of the framework processing device software is shown in Figure 2b. 

The interface driver(s) are used to establish and maintain a connection between the processing device and hardware. A graphical user interface is used to simplify the control of the framework functions and to display relevant information on the display unit of the processing device. An application programming interface is a part of the lower-level programming code that describes the functions or procedures used in the software of the framework processing device. It is used for integration with other programs or operating systems.

RF IC register controls are used to configure the registers of the RF transceivers and set wanted parameters. Graphical representation of data is used to visualise results of digital processing (measurement) of sent or received signals (data). State load/save function of the framework provides the possibility to boot or save a known configuration and could be used for quick verification, configuration loading, error checking. Data stream settings are used to set parameters of the communication between processing device unit and hardware, while FPGA code configuration is responsible for loading or updating firmware code. Signal clock configuration is used to set and control clock generator for the ADC/DAC and the parameters of the reference clock generation path. The framework controls part is used for general purpose hardware controls (switches, DAC values, etc.), Initialisation—to set the hardware of the framework to default settings and adjust the interface parameters, while Reset block is used to reset the hardware logic.

Firmware is loaded and updated using framework’s processing device software. Functionality depends on specific hardware configuration of the framework and can be easily upgraded (introduction of new features) or optimised (cost optimisation).

Structure of the framework’s firmware is shown in Figure 2c. As in the case of the processing device software, the interface driver(s) are used to establish and maintain a connection between the processing device and hardware. Core program contains implementation of functions that process digital signals, execute calibration algorithms, and control external hardware devices or extension boards. This piece of code uses a processor (for example, ARM family processor). The segment of the core program can be further subdivided into the smaller code blocks. GPIO controls are used to control general purpose I/O module (direction, data type). The security segment is used for data encryption and secure authentication between different devices. GNSS control is used to configure the global navigation satellite system module, send positioning, time synchronisation, reference pulse signals from it to the processing device or any other hardware. PCB switch network and pin header extension respectively control the analog signal (RF and IF) signal switches and configuration of the extension ports, while sensor control is used to receive sensor information and generate control signals for them. The calibration algorithm of the radio module’s transmitter and receiver (DC offset, quadrature imbalance, filter bandwidth, etc.) is implemented in the calibration algorithm’s block. Power management code implements management of power circuits and can be used for configuration of various power modes, voltage/current control/limitation, and power-down sequencing of individual systems. Memory control manages memory resources, while LED control provides visual indication of the hardware status.

FPGA firmware code defines its hardware functions (digital interfaces, codecs, etc.). It also implements RF IC data and RF IC control functions, interface configuration, and it can use video codec for data compression and processing.

## 3. Design of Network-in-a-Box

### 3.1. Design of Software Defined Radio Framework Hardware

**Figure 3:**
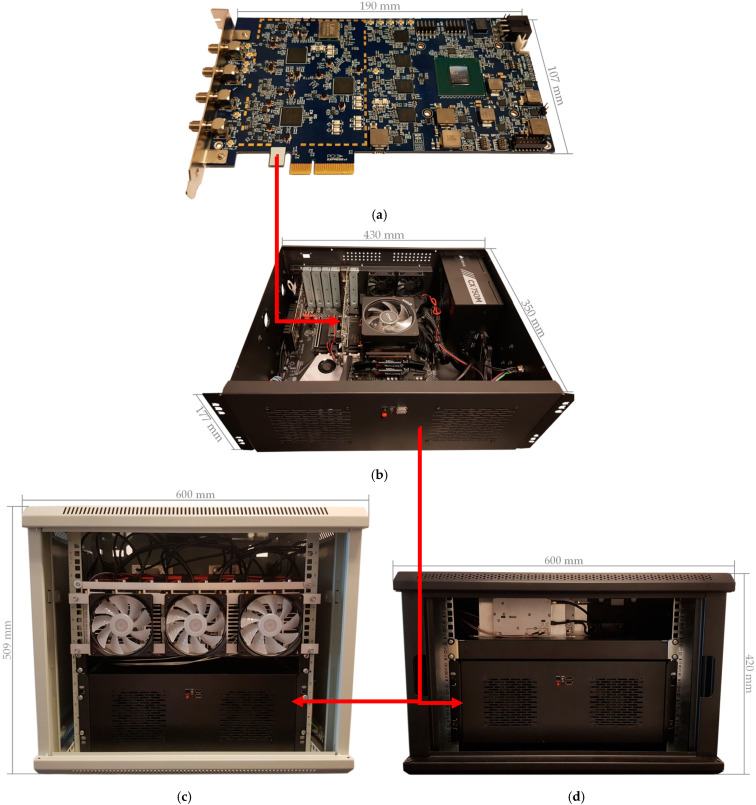
Network-in-a-box (NIB) structure: (**a**) software defined radio framework hardware; (**b**) general purpose processor core; (**c**) complete 4G carrier aggregation NIB (4G-CA) hardware with radio frequency front-end; (**d**) complete 5G standalone NIB (5G-SA) hardware with radio frequency front-end.

Based on the structure of the proposed SDR framework’s hardware presented in Section 2.1, a hardware of SDR-based framework (see Figure 3a) was developed based on Lime Microsystems technology in a single printed circuit board (PCB), with a length of 190 mm and a width of 107 mm. SDR framework uses three LMS7002M field programmable RF (FPRF) transceiver ICs. Two FPRFs are used to enable operation on two separate frequency bands simultaneously, with up to 100 MHz RF channels. The third FPRF IC can be used for spectrum monitoring, test signal generation and implementation of a calibration functionality. It can also be used as a dedicated receive chain to implement digital predistortion (DPD) solutions. 

Xilinx XC7A200T-2FBG676C FPGA from the Xilinx Artix-7 Family is used in SDR-based framework. Main blocks implemented in the FPGA are as follows:

- MicroBlaze soft microprocessor, which provides periphery controls.
- PCIe IP core, which provides data transfer between external host and FPGA through PCIe Gen2 interface.
- Receive-Transmit block, which is used to transfer IQ sample packets from/to LMS7002M transceiver chip and provide IQ sample synchronisation.
- LMS7002M interface block, which is used to send and receive data to/from LMS7002M IC.
- External ADC and DAC converter blocks, which is used to capture/transmit data from/to external ADCs and DACs.
- Synthesiser block, which provides required clock signals for receive-transmit blocks.

Table 1 provides utilisation of the main FPGA resources—slice look-up tables (LUTs), slice registers, block RAM (BRAM), digital signal processors (DSP). 

### 3.2. Design of Netowrk-in-a-Box Hardware

The designed NIB can be separated into two modular parts: GPP core and high power FEM. The designed SDR framework is a part of the GPP core in our NIB solution and is used as a highly configurable RF block in the RRH. The SDR framework is connected to the COTS x86 architecture GPP via a PCIe Gen2 interface.

GPP core is shown in Figure 3b. The main components are as follows: AMD Ryzen 3900× processor with base clock equal to 3.8 GHz; 16 GB, 3600 MHz memory; 512 GB solid-state drive; case size is 350 mm × 430 mm × 177 mm. Main hardware components of the GPP core are summarized in Table 2.

For performance evaluation, two NIB testbeds with different configurations were used:

1. 4G-CA: configuration supports operation on four different 4G FDD bands (1, 3, 7, 28).
2. 5G-SA: configuration supports operation on one 5G TDD band (78).

The other part of the RRH is the high power FEMs. Both NIB configurations use commercially available off-the-shelf FEMs with same RF specifications (only their count differs) which are presented in Table 3. FEM’s maximum modulated output power is 33 dBm, passband ripple—2.5 dB, noise figure ranges from 2 to 3.5 dB, error vector magnitude (EVM) ranges from 1.5 to 2 percent, and peak power consumption is 25 W. 

The 4G-CA NIB is housed in a standard 19-inch, 9U (509 mm × 600 mm × 450 mm) communication rack cabinet with a size of (see Figure 3c). Similarly, the 5G-SA NIB is housed in a lower, standard 19-inch, 7U (420 mm × 600 mm × 450 mm) rack cabinet because of its lower count of FEMs (see Figure 3d). As mentioned earlier, both GPP core and FEMs are modular and can be swapped in and out, upgraded to meet various NIB testbed use case scenarios.

## 4. Performance Evaluation of the Network-in-a-Box

### 4.1. Performance Evaluation Environment

Both 4G-CA and 5G-SA NIB configurations use Amarisoft software to implement functionality of the local RAN (eNB and gNB) and network core (EPC and 5GC). For control of the SDR framework, Lime Suite was used, which is an open-source collection of software supporting different hardware platforms and drivers for the FPRF.

4G-CA was configured for FDD operation, using a 20 MHz bandwidth for each carrier for all CA configuration test cases. Six different CA configurations used for 4G-CA NIB performance evaluation are presented in Table 4. 

In all test cases of the performance evaluation, the following common parameters apply for both NIB configurations:

- MIMO channel output power at antenna port is set to 33 dBm.
- The same MIMO omni directional antennas, mounted at a 2 m height, that work from 700 MHz to 3800 MHz with an average gain of 2 dBi were used at all ports.
- UEs were connected over-air, 5 m from the antennas and placed at a height of 1 m.
- 2 transmit (TX) and 1 receive (RX) antennas per band was used for DL and UL, respectively.

Results of the 4G-CA NIB single channel power measurement for CA configuration.

**Figure 4:**
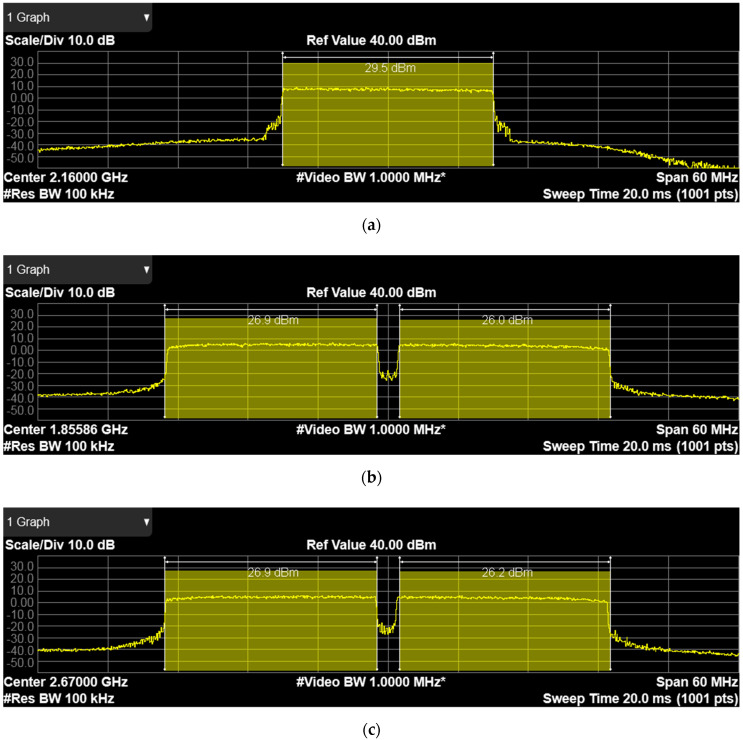

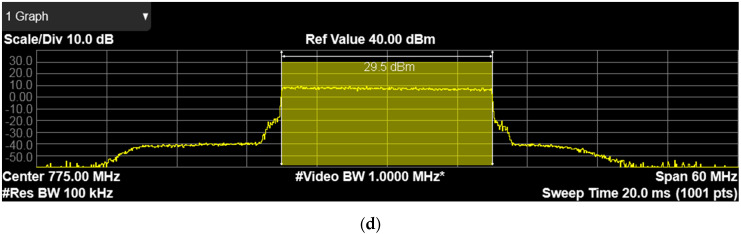
Results of the single channel power measurement for 4G carrier aggregation: (**a**) band 1, 20 MHz bandwidth, single carrier; (**b**) band 3, 40 MHz aggregated bandwidth, two contiguous carriers; (**c**) band 7, 40 MHz aggregated bandwidth, two contiguous carriers; (**d**) band 28, 20 MHz bandwidth, single carrier.

3C + 7C + 1A + 28A are shown in Figure 4. Measured channel power across all bands show a ±1 dBm variation from wanted levels. 

5G-SA was configured for TDD operation, using a 30 kHz subcarrier spacing. For all test cases of the bandwidth configuration, TDD pattern 1 with a 5 ms periodicity was used, having 7 DL and 2 UL slots, and 2 DL/UL symbols. Five bandwidth settings, ranging from 20 MHz to 100 MHz in 20 MHz intervals, were used for 5G-SA NIB performance evaluation. 

**Figure 5:**
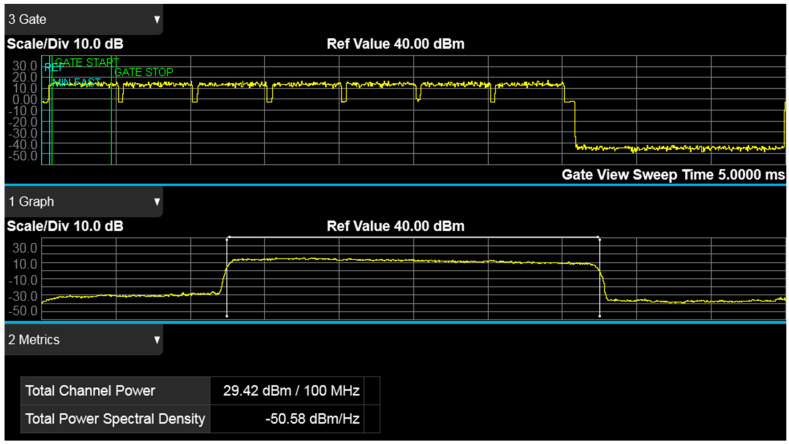
Time-gated spectrum analysis for a time division duplex with a 5 ms transmission periodicity and single-channel power measurement results for 5G standalone operation, band 78, 100 MHz bandwidth.

Result of the 5G-SA NIB single channel power measurement, when configured for 100 MHz bandwidth operation, is shown in Figure 5. 

Two Telit LM960A18 UEs and single Huawei P40 Pro UE were used for 4G-CA and 5G-SA NIB performance evaluation, respectively. A second Telit UE was used for 5 and 6 CA test cases to utilise the full bandwidth of the 4G-CA NIB.

### 4.2. Data Transfer Speed 

Iperf program was used to generate data traffic in both UL and DL directions during measurements of the data transfer speed. Measured 4G-CA transfer speeds are presented in Table 5. Average downlink speed ranges from 189 Mbps to 1023.8 Mbps when number of carriers is changed from 1 to 6. Average uplink speed ranges from 65.8 Mbps to 131.2 Mbps, when number of carriers is changed from 1 to 6. Maximum downlink and uplink speed is 1062 Mbps and 131.6 Mbps, respectively, when six carriers are used. Maximum uplink speed increases twice for 5 and 6 CA test cases due to the usage of a second Telit UE.

Measured 5G-SA transfer speeds are presented in Table 6. Average downlink speed ranges from 145.3 Mbps to 755.6 Mbps when bandwidth is changed from 20 MHz to 100 MHz. Average uplink speed ranges from 15 Mbps to 91.5 Mbps, when bandwidth is changed from 20 MHz to 100 MHz. Maximum downlink and uplink speed is respectively 765 Mbps and 92.6 Mbps, when 100 MHz bandwidth is set. 

**Figure 6:**
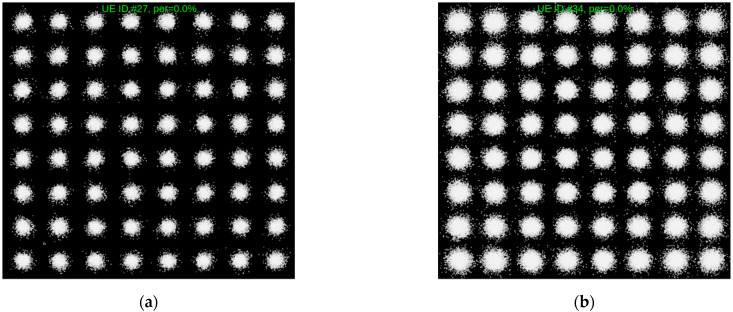
Uplink constellation diagram: (**a**) 4G carrier aggregation test case (5 carriers); (**b**) 5G standalone test case (100 MHz).

Figure 6 shows the uplink constellation diagram with a 0% packet error rate for the both 4G-CA and 5G-SA, when both NIBs are operating at a total aggregated bandwidth of 100 MHz.

### 4.3. Latency

Data of the round-trip packet latency over 4G and 5G links are respectively presented in Table 7 and Table 8. It should be noted that both eNodeB and gNodeB used a scheduling request period of 40 ms. Average ratio was measured from transfer of 100 packets. Relative change of the average packet latency over 4G link to the average packet latency over 5G link is presented in Table 9. It is seen from the data that average packet latency is lower over 5G link; it is up to −40.5% and −45.1% lower when the transmission of the packets is initiated from BTS and UE side, respectively. 

### 4.4. Central Processor Load

Load of the CPU for 4G and 5G links is shown respectively in Table 10 and Table 11. It is seen that in both 4G and 5G cases, CPU load by the eNodeB and gNodeB is proportional to occupied bandwidth. This is expected because sample rate and loading of the CPU increases with bandwidth due to the need of additional signal processing. In both test cases, gNodeB loads CPU less than eNodeB, so it is more effective from a processing perspective. 

It can be seen that EPC CPU load depends on UE count only and is independent of CA configuration when UE count is constant. This is understandable because UEs only use a single band for UL traffic. It can also be observed that 5GC CPU load in Test Case 3 is also fairly constant and bandwidth-independent. 

It can be concluded that in most NIB use case scenarios and under normal operating conditions (no simulated heavy traffic), EPC and 5GC software can be used locally without the need to move them on separate GPP systems, a practice often found in related works.

### 4.5. Power Consumption

Power consumption of the 4G-CA and 5G-SA NIBs respectively is presented in Table 12 and Table 13. Results of the power consumption do not include idle system power when network stack software is not running. Power consumption of idle 4G-CA and 5G-SA NIBs is respectively equal to 96 W and 90 W. Power consumption of the 4G-CA NIB increases in Test Case 1 and 2 when number of carriers increases. In Test Case 1, it changes from 38 W to 87 W, and in Test Case 2 it changes from 71 W to 216 W, when number of carriers increases from 1 to 6. Total power consumption when full DL and UL traffic is initiated changes from 100 W to 277 W, when number of carriers is changed from 1 to 6. Large power consumption changes in both Test Case 2 and 3 can be seen when additional band is used for higher CA configuration because of additional FEM usage.

Similarly, power consumption of the 5G-SA NIB increases in all three Test Cases when bandwidth increases. In Test Case 1, it changes from 42 W to 68 W, in Test Case 2 it changes from 72 W to 95 W, and in Test Case 3 it changes from 104 W to 130 W, when bandwidth increases from 20 MHz to 100 MHz. Lower total power consumption of the 5G-SA when full DL and UL traffic is initiated is mainly due to a lower count of FEMs. From a processing perspective, if at least three carriers are used for the 4G link, 5G is more power efficient. Power consumption for 80 MHz and 100 MHz bandwidths are identical due to same sample rate used in both cases. 

In both 4G-CA and 5G-SA configurations, total power consumption increase in Test Case 3, when comparing it to Test Case 2, is mainly due to increase of average channel power, hence the additional FEM power consumption under continuous DL traffic conditions. 

## 5. Conclusions

With an ever-increasing number of frameworks and projects of the RAN-oriented software stack becoming accessible to researchers and consumers, availability of the SDR-based RRH frameworks remains limited. Due to limited flexibility of available SDR-based RRH frameworks, available NIB solutions are restricted to single band operation in both 4G and 5G. Hence, solutions for expansion of the existing system capacity in existing infrastructure, for example usage of higher-level 4G CA, are not sufficiently explored and compared with 5G technology.

In this work, an open RAN-compatible, scalable, and highly flexible SDR-based RRH framework is proposed and designed. The main feature of the proposed framework is the ability of software and hardware reconfiguration with minimum changes to the overall framework. 

The performance of the proposed SDR framework is validated by creating two NIB-based testbeds that can operate in multiband multicarrier 4G or 5G SA configurations capable of RF operation from 700 MHz to 3800 MHz. The designed NIBs are separated into two modular parts—COTS-based GPP core and high-power FEM—capable of providing a maximum channel output power of 33 dBm at all bands. 

For 4G-CA, when 1 UE is active, a maximum of 883 Mbps DL and 65.8 Mbps UL data transfer speed is achieved when an aggregated bandwidth of 100 MHz is used. Under the same aggregated bandwidth conditions, 5G-SA achieves a 762 Mbps DL and 92.6 Mbps UL data transfer speeds. If six carriers are used in the 4G NIB, 1062 Mbps downlink data transfer speed can be achieved.

Average packet latency is lower in the 5G-SA; it is up to 40.5% and 45.1% lower when the transmission of the packets is initiated the from BTS and UE side, respectively, when comparing with the 4G-CA.

CPU load by the eNodeB and gNodeB is proportional to occupied bandwidth, but under the same aggregated DL bandwidth conditions, gNodeB is more effective from a processing perspective and loads CPU less than eNodeB. When only 1 UE is active, the EPC CPU load is up to four times lower than the 5GC under same aggregated bandwidth conditions.

FEMs are one of the main power consumers of a high-power NIB solutions. For 5G SA, power consumption at different bandwidth configurations is determined by CPU efficiency because power consumption of the FEM module is nearly constant. For 4G-CA, usage of higher number of carriers not only increases CPU power draw but also multiplies the FEM power draw due to the need of additional modules to support operation at different bands.

As a future step, based on the validated SDR framework, a new FPRF IC is planned to be developed. The new IC will support most of the RF and digital features of current SDR framework in a single package.

## Figures and Tables

**Table 1:** Utilisation of the main FPGA resources.

Resource	Available	Used	Utilisation, %
Slice LUTs	133800	75551	56.47
LUT as Logic	133800	72875	54.47
LUT as Memory	46200	2676	5.79
Slice Registers	267600	48805	18.24
Block RAM	365	117	32.05
DSP	740	122	16.49

**Table 2:** Hardware summary of general-purpose processor core of the network-in-a-box.

Computer	Processor	AMD Ryzen 3900×, 3.8 GHz
	Memory	2 × 8 GB, 3600 MHz
	Storage	512 GB SSD
Software Defined Radio Framework	RF transceiver	LMS7002M
	FPGA	XC7A200T-2FBG676C
	MIMO	2 × 2 per FPRF IC, 4 × 4 total
	Connectivity	PCIe ×4 (Gen2)

**Table 3:** Specification of the Front-End Module.

Parameter	Value
Modulated output power, dBm	33
Passband ripple, dB	2.5
Noise figure, dB	2–3.5
Error vector magnitude,%	1.5–2
Peak power consumption, W	25

**Table 4:** Carrier aggregation configurations used during 4G-CA performance evaluation.

Carrier Aggregation Configuration	Number of Carriers	Frequency Band	DL Center Frequency, MHz	Bandwidth, MHz	Total Bandwidth, MHz
3A	1	3	1846	20	20
3C	2	3	1856	20 + 20	40
3C + 7A	3	3	1856	20 + 20	60
		7	2660	20	
3C + 7C	4	3	1856	20 + 20	80
		7	2670	20 + 20	
3C + 7C + 1A	5	3	1856	20 + 20	100
		7	2670	20 + 20	
		1	2160	20	
3C + 7C + 1A + 28A	6	3	1856	20 + 20	120
		7	2670	20 + 20	
		1	2160	20	
		28	775	20	

**Table 5:** 4G carrier aggregation transfer speed.

Number of Carriers	Total Bandwidth, MHz	Maximum DL Speed, Mbps	Average DL Speed, Mbps	Maximum UL Speed, Mbps	Average UL Speed, Mbps
1	20	189.0	189.0	65.8	65.8
2	40	373.0	367.3	65.8	65.7
3	60	549.0	543.2	65.8	65.6
4	80	719.0	700.2	65.8	65.1
5	100	883.0	856.6	131.6	131.2
6	120	1062.0	1023.8	131.6	131.2

**Table 6:** 5G standalone transfer speed.

Bandwidth, MHz	Maximum DL Speed, Mbps	Average DL Speed, Mbps	Maximum UL Speed, Mbps	Average UL Speed, Mbps
20	146.0	145.3	15.3	15.0
40	310.0	306.6	34.3	34.1
60	476.0	472.2	53.5	53.1
80	636.0	633.0	69.3	68.7
100	762.0	755.6	92.6	91.5

**Table 7:** Round-trip packet latency over 4G.

Ping Source		BTS to UE				UE to BTS			
Ping Period, ms		1	10	100	1000	1	10	100	1000
Packet size = 100 bytes	Minimum latency, ms	16.0	21.8	17.9	18.7	14.0	13.0	19.0	21.0
	Average latency, ms	34.0	31.1	38.1	39.5	32.0	44.0	37.0	47.0
	Maximum latency, ms	48.9	55.3	58.1	57.1	59.0	65.0	58.0	60.0
	Standard deviation, ms	9.4	8.3	11.7	11.2	13.7	11.0	7.8	11.1
Packet size = 1000 bytes	Minimum latency, ms	22.1	23.9	26.8	26.2	22.0	25.0	28.0	22.0
	Average latency, ms	31.4	41.2	45.8	46.0	51.0	48.0	42.0	49.0
	Maximum latency, ms	52.1	68.1	65.9	66.1	67.0	67.0	68.0	65.0
	Standard deviation, ms	7.2	13.9	11.3	11.4	10.8	9.4	9.5	11.7

**Table 8:** Round-trip packet latency over 5G.

Ping Source		BTS to UE				UE to BTS			
Ping Period, ms		1	10	100	1000	1	10	100	1000
Packet size = 100 bytes	Minimum latency. ms	9.6	11.9	12.0	12.0	13.0	15.0	14.0	16.0
	Average latency. ms	23.4	22.7	26.4	30.1	27.0	27.0	27.0	27.0
	Maximum latency. ms	44.2	40.0	40.3	43.7	42.0	44.0	46.0	49.0
	Standard deviation. ms	8.5	6.3	7.4	7.8	6.9	7.8	6.7	6.9
Packet size = 1000 bytes	Minimum latency. ms	9.8	13.8	20.7	21.9	24.0	25.0	24.0	23.0
	Average latency. ms	23.4	24.5	30.2	33.1	28.0	29.0	31.0	31.0
	Maximum latency. ms	40.3	40.5	49.0	48.5	43.0	45.0	46.0	47.0
	Standard deviation. ms	7.8	7.9	6.1	6.2	4.1	4.3	4.9	6.0

**Table 9:** Relative change of the average packet latency over 4G to 5G.

Ping Source	BTS to UE				UE to BTS			
Ping Period, ms	1	10	100	1000	1	10	100	1000
Relative change for 100 bytes,%	−31.2	−27.0	−30.7	−23.8	−15.6	−38.6	−27.0	−42.6
Relative change for 1000 bytes,%	−25.5	−40.5	−34.1	−28.0	−45.1	−39.6	−26.2	−36.7

**Table 10:** Load of the CPU running 4G carrier aggregation.

Number of Carriers	Total Bandwidth, MHz	eNodeB CPU Load, Test Case 1 ^1^,%	eNodeB CPU Load, Test Case 3 ^2^,%	EPC CPU Load, Test Case 3 ^2^,%	Total CPU Load, Test Case 3 ^2^,%
1	20	5.3	9.0	3.3	12.3
2	40	12.7	19.0	3.3	22.3
3	60	17.8	27.3	3.3	30.7
4	80	23.6	35.8	4.2	40.0
5	100	30.5	49.2	7.8 ^3^	57.0
6	120	36.7	63.3	7.8 ^3^	71.2

^1^ eNodeB and EPC active, FEM disabled. ^2^ eNodeB and EPC active, FEM enabled, full DL and UL traffic. ^3^ CPU load doubles due to second Telit UE.

**Table 11:** Load of the CPU running 5G standalone.

Bandwidth, MHz	gNodeB CPU Load, Test Case 1 ^1^,%	gNodeB CPU Load, Test Case 3 ^2^,%	5GC CPU Load, Test Case 3 ^2^,%	Total CPU load, Test Case 3 ^2^,%
20	4.5	6.9	16.7	23.6
40	8.8	13.9	16.7	30.6
60	14.4	21.7	17.5	39.2
80	18.6	30.0	17.5	47.5
100 ^3^	20.0	30.8	18.1	48.9

^1^ gNodeB and 5GC active, FEM disabled. ^2^ gNodeB and 5GC active, FEM enabled, full DL and UL traffic. ^3^ Sample rate is same as in 80 MHz bandwidth case.

**Table 12:** Power consumption of the 4G-CA network-in-a-box.

Number of Carriers	Total Bandwidth, MHz	Power Consumption, Test Case 1 ^1^, W	Power Consumption, Test Case 2 ^2^, W	Power Consumption, Test Case 3 ^3^, W
1	20	38	71	100
2	40	50	79	107
3	60	58	138	163
4	80	66	143	168
5	100	74	179	231
6	120	84	216	277

^1^ eNodeB and EPC active, FEM disabled. ^2^ eNodeB and EPC active, FEM enabled. ^3^ eNodeB and EPC active, FEM enabled, full DL and UL traffic.

**Table 13:** Power consumption of the 5G-SA network-in-a-box.

Bandwidth, MHz	Power Consumption, Test Case 1 ^1^, W	Power Consumption, Test Case 2 ^2^, W	Power Consumption, Test Case 3 ^3^, W
20	42	72	104
40	54	81	112
60	60	90	120
80	68	95	130
100 ^4^	68	95	130

^1^ gNodeB and 5GC active, FEM disabled. ^2^ gNodeB and 5GC active, FEM enabled. ^3^ gNodeB and 5GC active, FEM enabled, full DL and UL traffic. ^4^ Sample rate is same as in 80 MHz bandwidth case.
